# Supplementary material for: Optical and Material Characteristics of MoS2/Cu2O Sensor for Detection of Lung Cancer Cell Types in Hydroplegia
Source: Int J Mol Sci. 2022 Apr 25;23(9):4745. doi: 10.3390/ijms23094745 (PMC9101548; doi:10.3390/ijms23094745)
Supplement: Supplementary file 1 [file ijms-23-04745-s001.zip › ijms-1689894-supplementary.pdf]

Article

Optical and Material Characteristics of MoS_2/Cu_2O Biosensor for Detection of Lung Cancer Cell Types in Hydroplegia: Supplementary Document

Arvind Mukundan [1], Shih-Wei Feng [2], Yu-Hsin Weng [1], Yu-Ming Tsao [1], Sofya B. Artemkina [3],4, Vladimir E. edorov [3],4, Yen-Sheng Lin [5], and Hsiang-Chen Wang [1], ∗.

1 Department of Mechanical Engineering and Advanced Institute of Manufacturing with High tech Innovations, National Chung Cheng University, 168, University Rd., Min Hsiung, Chia Yi 62102, Taiwan
2 Department of Applied Physics, National University of Kaohsiung, 700 Kaohsiung University Rd., Nanzih District, Kaohsiung 81148, Taiwan; swfeng@nuk.edu.tw
3 Nikolaev Institute of Inorganic Chemistry, Siberian Branch of Russian Academy of Sciences, Novosibirsk 630090, Russia; artem@niic.nsc.ru (S.B.A.); fed@niic.nsc.ru (V.E.F.)
4 Department of Natural Sciences, Novosibirsk State University, 1, Pirogova str., Novosibirsk 630090, Russia
5 Department of Electronic Engineering, I-Shou University, No.1,Sec. 1, Syuecheng Rd.,Dashu District , Kaohsiung City 84001, Taiwan; yslin@isu.edu.tw
* Correspondence: hcwang@ccu.edu.tw

Abstract: This document provides supplementary information to "The Optical and Material Characteristic Study of MoS_2/Cu_2O Heterojunction Surface: Supplementary Document". Section 1 describes about the Characteristics, Synthesis method of Cu_2O. Section 2 explains in detail about the apparatus and intruments used in this study. Section 3 talks about the list of substrates, organic solvents, gases, and chemicals used to grow Cu_2O and MoS_2. Section 4 describes the results of Cu_2O electrochemical growth under different parameters while the final section describes the CVD growth results of MoS_2

Keywords: Photoelectrochemical, Chemical Vapor Deposition (CVD), Molybdenum Disulfide (MoS_2), Cuprous Oxide (Cu_2O), Positive Oxide Trap State, DNA, Biosensor

1. Introduction to Cu_2O

1.1. *Material characteristics of Cu_2O*

Cu_2O (Cuprous Oxide) is one of the first semiconductors to be studied. It is used to make antifouling paints, bactericides, ceramic colorants, in manufacturing of various copper salts, analytical reagents, etc. This project has successfully adopted electrochemical deposition system to grow Cu_2O on an ITO (Indium Tin Oxide) surface substrate, and the thickness of the Cu_2O film can be adjusted according to the deposition time. Cuprous oxide is the oxide of monovalent copper and its appearance is a highly symmetric cubic crystal structure from red to macro brown. Its molar mass is 143.09g/mol, density is $6.0g/cm^3$, and melting point is 1185° C. The refractive index is 2.705. It is generally insoluble in water and organic solvents, but soluble in dilute hydrochloric acid, dilute sulfuric acid, ammonium chloride solution, and will be slowly oxidized to copper oxide in humid air. It is a P-type semiconductor with a direct energy gap of 1.8-2.2eV. Due to the characteristics of Cu_2O in the visible light band of high absorption rate and poor transmittance, and the unbonded exciton binding energy has a bonding energy of 150meV. The conversion of photons into excitons occurs in the visible wavelength range, and the direct band gap semiconductor has no dipole moment, which can extend the survival time of excitons. Therefore, there is considerable potential for solar cells, sensors and other related research, which have been widely used by scientists in the field of discussion and research. The physical properties of cuprous oxide mentioned above are summarized as shown in Table S1.

Two-dimensional material	Cuprous oxide (Cu_2O)
Mohr mass	143.09g/mol
Density	6.0g/cm³
Melting point	1232°C
Refractive index	2.705
Bandwidth	1.8-2.2eV (direct energy gap)
Solubility	insoluble in water, soluble in acid (except HCl)
Crystal structure	cubic crystal

Table S1: Physical properties of Cu_2O

1.2. Synthesis method of Cu_2O

There are many kinds of Cu_2O synthesis methods, such as thermal oxidation method, electrochemical deposition method, chemical vapor deposition method, spray pyrolysis method, wet chemical solution method, reactive sputtering method, anodic oxidation method and molecular beam epitaxy method. Most of its researches uses thermal oxidation method, which has the advantage of high carrier mobility. However, it requires high-temperature equipment, which makes the cost relatively high, and the film characteristics of reactive sputtering are slightly lower than other process methods. On the other hand electrochemical deposition method consists of a simple process, low cost, high reproducibility, and large area uniform deposition. This method can effectively control the crystal shape and crystal lattice direction by changing the potential and the pH value of the solution. There are also no restrictions on the choice of substrate thickness and size, so that it can be widely used in different researches.

1.3. Obtaining the layers of MoS_2

The most common research methods used to obatain the layers of MO_2 are Mechanical Exfoliation Method, Chemical Vapor Deposition, Electrochemical Lithiation Process and Sulfurization of MoO_3. Among them, the chemical vapor deposition method can efficiently grow a large area and uniform single-layer MoS_2 film, which is also the most commonly used process method is employed in this current research.

1.3.1. Chemical vapor deposition (CVD)

The use of CVD can control high-quality MoS_2 morphology, number of layers and uniform and large-area thin films. For the appearance of MoS_2, the shape of the area on the substrate will evolve with the ratio of Mo to S (1:>2, 1:2, and 1:<2), and through SEM, Raman spectra, AFM and PL. The test results confirmed that the CVD method can grow a uniform and highly crystalline single-layer MoS_2 film.

2. Instrument Specifications

2.1. Electrochemical Deposition system

The two electrodes are connected to a DC power supply as the cathode and anode during electrochemical deposition. The working electrode is an ITO glass substrate and the counter electrode is a graphite rod. The bracket designed in this experiment can grow three Cu_2O films in one experiment. The ITO glass substrate and graphite rod are placed in a beaker electrolyzer for electrochemical method. The temperature is controlled at 60°C and the temperature holding time is 30 minutes.

2.2. Micro-Raman Spectroscopy

Raman spectra is a method that can effectively analyze the molecular structure, molecular vibration mode, and rotation mode to study the determination of the crystal lattice and the position of molecules or chemical bonds. Raman scattering uses the interaction of excitation light and matter to produce inelastic scattering. The general laser range is visible light, near-infrared light or near-ultraviolet light. In order to understand the number of layers of MoS_2 film, Raman spectrometer is used. The model

of the Raman microscope used in this experiment is HORIBA XploRA ONE. The Raman microspectrometer uses a 532nm laser to enter the sample. The photons in the laser will collide with the molecules in the sample material to generate Raman scattered light, which is received by the instrument After measuring the molecular vibration. For example, the MoS_2 film, the E_{12g} and A_{1g} vibration modes are the main signals for the judgment of MoS_2. The two vibration modes are highly dependent on the thickness of MoS_2. When the k value of the subtraction of E_{12g} and A_{1g} peaks is less than 20cm^{-1}, the analyzed MoS_2 is regarded as a single-layer structure.

2.3. Micro-Raman Micro-PL spectrometer

Micro-Raman and low-light excitation fluorescence spectrometers are Horiba Jobin Yvon LabRAM HR systems. It has a powerful non-destructive spectrum measurement function, which can separately analyze the light-excited fluorescence spectrum and Raman spectrum. The light source is 633nm, 532nm and 325nm lasers. The Raman spectrum ranges from 150 to 5000cm^{-1}. It is capable of two-dimensional image scanning, low temperature (77 - 300 K), polarization measurement, and the sample can be measured without additional complicated pre-processing. From the measurement of light-excited fluorescence spectra, important information such as transmission path, energy gap size, band structure, doping impurity types, carrier transition behavior, composition of compounds, size effects and carriers in nanomaterials can be obtained. The measurement of Raman spectroscopy can be applied to different fields. Many occasions that require non-destructive, microscopic, chemical analysis and imaging analysis are involved. Raman can be used to quickly detect its chemical composition and structure. In addition to being used as a basis for judging the structure, composition and quality of materials, it is also often used in the fields of carbon materials, semiconductor materials, pharmaceuticals and cosmetics, life sciences, geology, and mineralogy. The PL measurement results in this study uses a 532nm laser as the excitation light source to measure the PL of a single-layer MoS_2 film. The measurement results show that a strong signal is generated at the wavelength of 667nm, and the energy is 1.84eV after conversion. It is the energy gap of single-layer MoS_2.

2.4. Optical Microscope

Optical microscope (OM) uses an optical lens to produce image magnification effect. The light incident from the object is magnified by the objective lens and eyepiece of the optical system. There are 5X, 10X, 40X and 100X on the objective lens plate while the magnification can be changed as required. According to the design of condenser and objective, optical microscopes can be divided into reflective and transmissive types according to different samples. Reflective microscopes are generally used for opaque samples. After the light is irradiated on the object, the light reflected by the object enters the microscope to obtain an image, which is mostly used to observe solids, materials, etc. The transmission microscope is for the transparent or very thin state of the sample itself, so that the light can enter the microscope from the sample. The image is generally used to observe biological tissues. The stage is the platform that carries the sample. There is an adjustable aperture below it. When the light is dark, you can adjust the aperture to choose a larger aperture.

2.5. Scanning Electron Microscope (SEM)

The high-resolution cold field emission scanning electron microscope (Cold field Emission Scanning Electron Microscope, FESEM) model HITACHI-S4800-I is used in the study. Compared with the optical microscope the magnification is about 1500 times. The magnification of the SEM can reach more than 10,000 times and the depth of field is large. It is mainly used to observe the microstructure of the sample surface and section. The principle of SEM is to apply an external electric field and accelerating voltage to make the electron gun generate a high-energy electron beam through the principle

of thermal dissociation or field emission, which is focused on the sample through an electromagnetic lens. The scanning coil deflects the electron beam on the sample surface to create a two-dimensional space scanning. When the electron beam interacts with the sample, it will emit various signals, such as secondary electrons, backscattered electrons, absorbed electrons, etc. Generally, the electron beam is focused on the sample, and the electrons on the sample surface will collide elastically and in-elastically. The information with the surface topography is received and imaged by the secondary electron detector, so the image of the unevenness of the sample surface is obtained. The SEM used in this experiment is HITACHI-S4800-I with a maximum magnification of 220K. Before observing the surface morphology, the sample must be kept dry to avoid affecting the vacuum degree. At the same time, Cu_2O is a non-conducting material. To have a clearer image, the surface of the sample needs to be plated with platinum first to avoid the concentration of charges on the surface of the sample, which will affect the clarity of the sample, and MoS_2 is used for subsequent transfer, and the surface is not plated with platinum to observe the morphology.

2.6. Atomic Force Microscope (AFM)

Atomic force microscope (AFM) is a nano-level high-resolution scanning probe technology. Through observation atoms and molecules can be visually seen. The micro cantilever is used to sense and amplify the Van der Waals force between the tip of the cantilever and the atoms of the sample under test to further show the surface characteristics of the sample. When the atom is very close to each other, the repulsive force of the electron cloud is greater than the attractive force between the nucleus and the electron cloud, making the entire net force behave as a repulsive force. On the contrary, if two atoms are separated by a certain distance, the repulsive force of the electron cloud between the two will be less than the attraction between the nucleus and the electron cloud of each other. The application of AFM technology is quite simple, no additional processing (such as platinum plating, baking) is required for the sample, and the damage to the sample is less than other detection technologies, and it can also be measured in a variety of environments (such as air, liquid). Live cells can also be dynamically observed, but unlike electron microscopes, AFM can provide three-dimensional images, and AFM imaging has a smaller range and slower speed than SEM. AFM measurement can be divided into three types of operations based on the distance between the probe and the sample: contact, non-contact, and percussion. The measurement system is a non-contact measurement when the distance between the tip of the atom and the atom on the sample surface.

2.7. X-ray Diffraction

X-ray diffraction analysis is a non-destructive analysis technique used to detect the characteristics of crystal materials. This model used in this study is Bruker Smart APEX. It provides structure and phase crystal orientation and parameter analysis (such as average particle size, crystallinity, tension, crystal defects, etc.). The X-ray diffraction peak is the constructive monochromatic light diffracted by the crystal lattice plane of the sample at a specific angle. Produced by interference, the peak intensity is determined by the distribution of atoms in the lattice. Generally, X-ray sources used in XRD are divided into two types: (1) photons generated by electron acceleration; (2) X-rays emitted by electrons with high energy impacting the target material to transfer energy. This X-ray can be divided into characteristic X-ray and continuous X-ray. The source of characteristic X-ray is high-energy electrons hitting the target. The incident electrons excite the inner orbital electrons of the target atoms, and the outer orbital electrons fill the inner layer. The energy of electrons transitioning between the two orbitals is released in the form of X-rays. Different atoms and different orbital combinations have different energies. Continuous X-rays do not involve energy level conversion. The source is after electrons enter the target. The speed drops rapidly and energy is released. Crystals can

be used as X-ray gratings. The coherent scattering of these large numbers of atoms, ions or molecules will cause light interference, which will affect the intensity of scattered X-rays to increase or decrease. Due to the superposition of a large number of atomic scattered waves, the beams that interfere with each other to produce the highest intensity are called X-ray diffraction lines. To meet the diffraction conditions, the Bragg formula can be applied: 2dsin=λ, using X-rays of known wavelength to measure the angle θ, thereby calculating the interplanar spacing d, which is used for X-ray structure analysis. The other method is to use a crystal with a known d to measure the θ angle to calculate the wavelength of the characteristic X-ray, and then to find out the elements contained in the sample from the existing data.

2.8. Multiphoton excitation microscope

This double-frequency (SHG) image is a micro-nanocenter-multiphoton excitation scanning microscopy system, composed of Chameleon Vision II titanium sapphire femtosecond laser (width 140fs) and its optical parameters oscillator are used as the excitation light source. The wavelength range can be from 680nm to 1600nm. The fast galvanometer and Z-axis automatic stage can be used for fast large-area scanning and 3D scanning. The rear end is focused and collected by a ZEISS microscope. The objective lens includes 10×/0.45, 20×/ 0.8, 40×/1.0. The sample is excited by pulsed laser to produce two-photon, three-photon absorption and emission, and double-frequency and triple-frequency signals. The fluorescent signals can be distinguished by different filters: 420 /10 nm, 450/70 nm, 531/40 nm, 600/37 nm, 660/52 nm. The optical signal is converted into a digital TTL signal, and the fluorescent image obtained by scanning is displayed. The absorption is low, and the scattering effect can be reduced to facilitate the observation of samples with high scattering properties. The high energy of the short-wavelength continuous laser used in the traditional conjugate focus microscope is likely to cause photobleaching, burning, and photochemical toxicity of the excited part of the sample to cause cell necrosis. Therefore, it is not suitable for long-term observation of living cells. The multiphoton microscope only images at the focal point, so pinhole filtering is no longer used. Using the principle of nonlinear optics, the planar scanning resolution is about 300nm or less, and the vertical resolution is increased to less than 1μm, and the sample absorbs long-wavelength infrared light. It can reduce the scattering effect and is conducive to the observation of high-scattering samples, so it has a better observation depth than the general short-wavelength continuous laser system. In biomedical applications, the received signal band can be selected according to different fluorescent dyes, and then scanned The fluorescent image is displayed for further analysis and research.

2.9. Transmission Electron Microscope (TEM)

The high-resolution transmission electron microscope (TEM) model is PHILIPS CM-200 TWIN which can observe the morphology of the material and the fine appearance, lattice structure and defects inside the material. The electron beam operating at 20-200KV has a wavelength of 0.0251nm and a point resolution of about 0.27nm, which is suitable for observing atomic distance images. By using a high-energy electron beam (100KeV-1MeV) to penetrate the material to be tested, and the internal structure of the material will produce elastic scattered electrons and inelastic scattered electrons. The size of the scattering angle is related to the density and thickness of the object, because the electrons can be collected and elastically scattered to obtain the information of the atomic arrangement; the information of the material composition and bonding can be obtained by inelastic scattering, and then the contrast of light and dark can be obtained through the combination of different optical lenses. After obtaining the contrast of light and dark images through different optical lens combinations, they are displayed by a CCD imaging system. In addition, an X-ray energy spectrum dispersion analyzer (EDS)

and an electron energy loss spectrometer (EELS) can be added to detect the difference in the material.

3. List of substrates, organic solvents, gases, and chemicals used to grow Cu_2O and MOS_2

List of substrates, organic solvents, gases, and chemicals used to grow Cu_2O are given in the Table S1 and while the List of substrates, organic solvents, gases, and chemicals used to grow MOS_2 are given in the Table S2.

Drug Name	Specification	Manufacturer	Agent Vendor
ITO Substrate	Size: 2cm*2cm	Ruilong Optoelectronics	Ruilong Optoelectronics
Methanol	Purity: 99.9%	Shimajiu Pharmaceutical	Rishun Instruments
Acetone	Purity: 99.9%	Shimajiu Pharmaceutical	Rishun Instruments
Isopropanol	Purity: 99.9%	Sigma-Aldrich	Youhe Trading
Pentahydrate and copper sulfate	Purity: 98+%	Alfa Aesar	Jingming Chemical
DL-Lactic acid	Purity: 85%	TEDIA	Jingming Chemical
Sodium Hydroxide	Purity: 98+%	Sigma-Aldrich	Youhe Trading
De-ionized water	Purity: 99.9%	Rephile	Yushun Instruments
High Pressure Nitrogen	Purity: 99.9%	Lianhua Gas	Lianhua Gas

Table S2: List of substrates, organic solvents, gases, and chemicals used to grow Cu_2O

Drug Name	Specification	Manufacturer	Agent Vendor
SiO_2	Size: 4 inches	Yimei Materials	Yimei Materials
S powder	99.98%	Weiss Enterprise	Weiss Enterprise
MoO_3 Powder	99.95%	Weiss Enterprise	Weiss Enterprise
Acetone	Purity: 99.9%	Shimajiu Pharmaceutical	Rishun Instruments
Sodium Hydroxide	Purity: 98+%	Sigma-Aldrich	Youhe Trading
PMMA A5	molecular weight:950	Asia Pacific International Electronics	Asia Pacific International Electronics
Nitric acid	Purity:68-69%	CHONEYE	Yushun Instruments
HCl	Purity: 37%	FLUKA	Jingming Chemical
De-ionized water	Purity: 99.9%	Rephile	Yushun Instruments
High Pressure Nitrogen	Purity: 99.9%	Lianhua Gas	Lianhua Gas

Table S3: List of substrates, organic solvents, gases, and chemicals used in MoS_2

4. The results of Cu_2O electrochemical growth under different parameters

When using the electrochemical method to grow Cu_2O thin films, the composition, concentration, temperature, pH value and other parameters of the solution will affect the deposition result. Among them, the sources of copper ions in the electrochemical solution are mainly copper sulfate, copper nitrate and copper acetate. The acid radicals of the drug dissolved in water will affect the electrochemical kinetics behavior during deposition. In order to obtain high-quality Cu_2O films, copper sulfate was selected as the copper source in this experiment, and Cu_2O was deposited at a concentration of 0.4M. Generally speaking, the Cu_2O deposition environment has a high sensitivity to the pH value, so we can use the characteristics of the pH value to indirectly control its preferred growth lattice plane as shown in Figure S1.

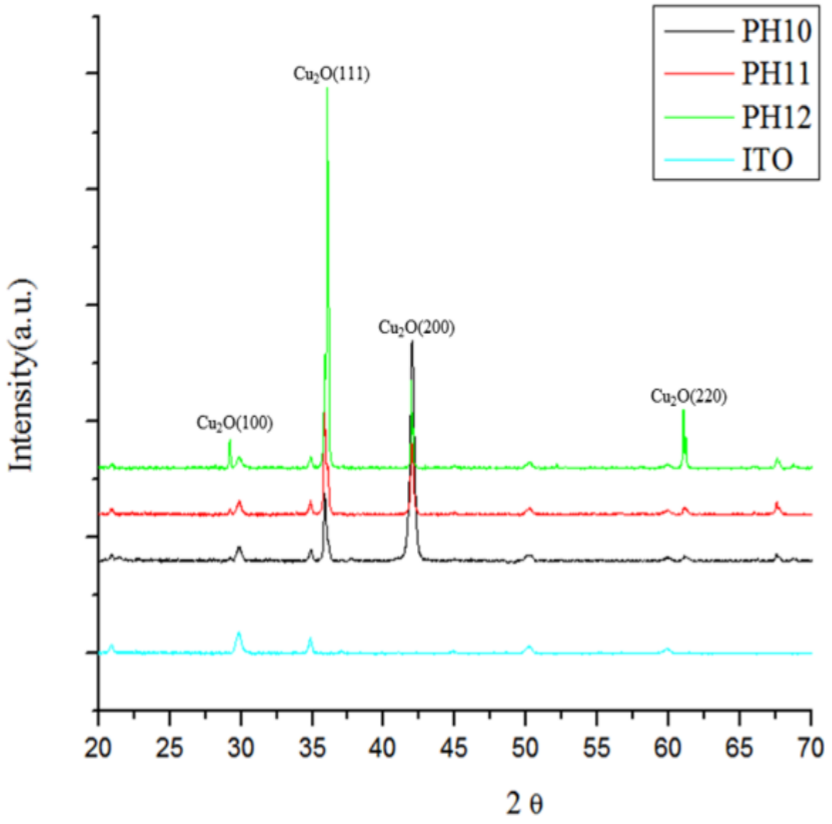

Figure S1. The XRD pattern of Cu_2O films grown under different deposition environment pH values

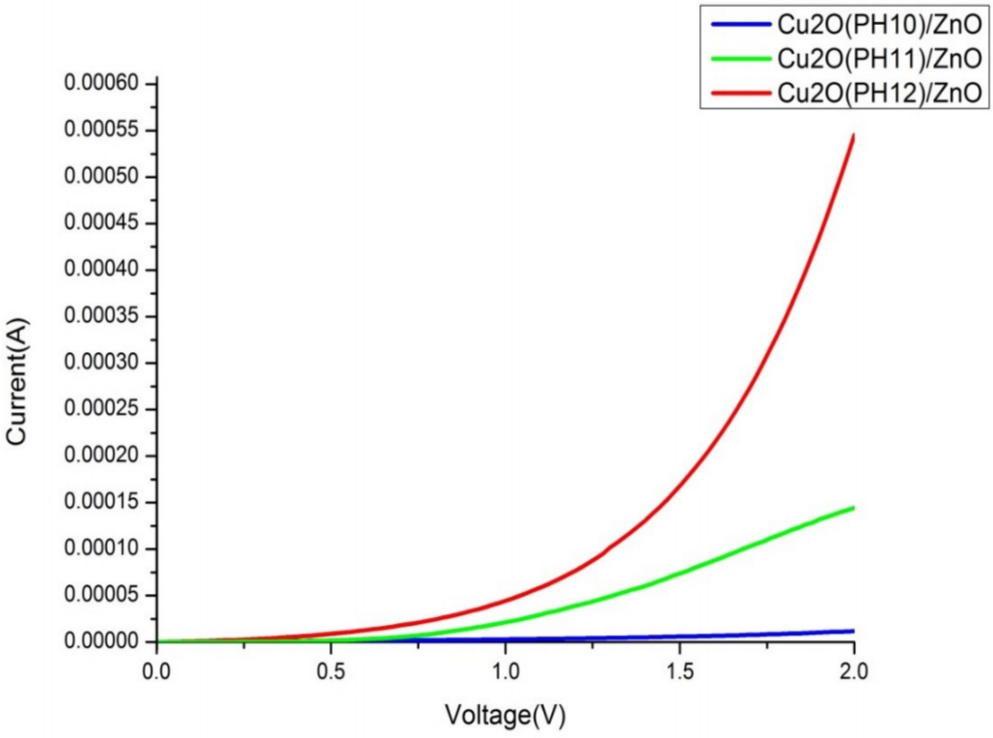

Figure S2. The I-V characteristic curve of samples PH10, PH11, PH12

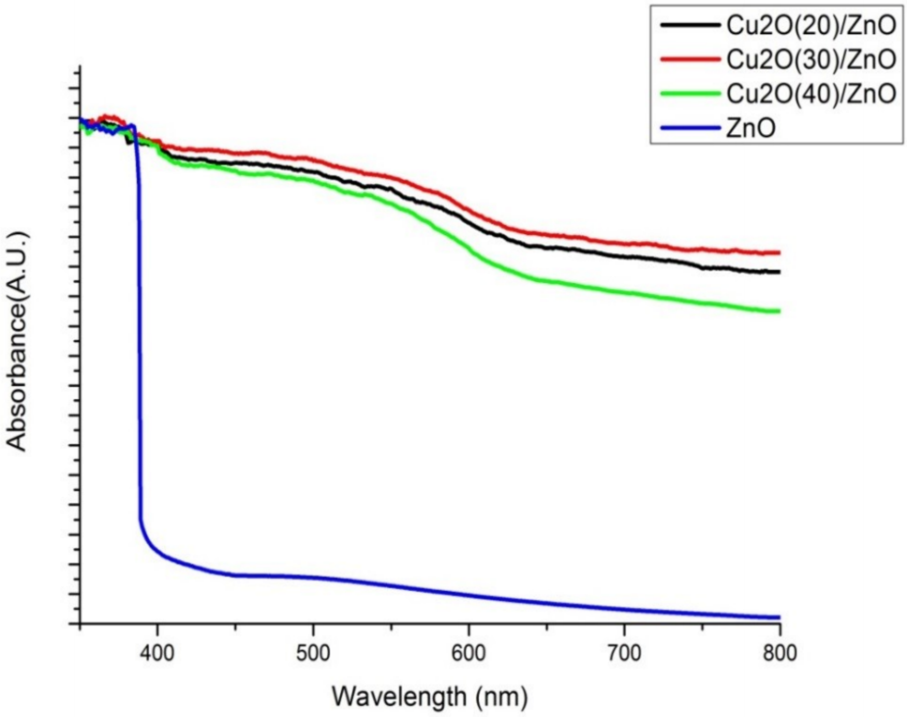

Figure S3. The absorption spectra of Cu_2O deposited in different thicknesses

From the XRD diagram, it can be observed that Cu_2O diffraction peaks appear at 2=29.6°, 36.5°, 61.5°, and 73.7°. By comparing with JCPDS Card, Cu_2O is in (110), (111), (200), (220).) And (311). The results show that the optimal growth of the (111) or (200) lattice plane can be effectively controlled by the pH value. According to the signal results of the samples of PH10, PH11, and PH12, it is found that as the pH value of the deposition environment increases, the strength of the (111) lattice plane gradually increases. This lattice plane has better light absorption ability, and it is found in (200) there is also a significantly stronger signal in the direction of the lattice, and this lattice plane has good electrical properties and corrosion resistance. Continuing the sample-entry electrical measurement of the above XRD measurement, as the electrochemical method to select the best pH value of Cu_2O, as shown in Figure S2.

It can be observed from the figure that as the pH value of the deposition environment increases, the measured electrical properties tend to be better. This is because the pH value depends on the preferred orientation of the crystal lattice during Cu_2O deposition. When the PH value is 10, it is possible that the crystal lattice contains more defects and the lattice signal is weaker, which reduces its electrical properties; when the pH value increases, the signal in the preferred direction of the (111) lattice also rises. The IV characteristic measurement found that it has relatively good electrical properties when the PH value is 12, so the subsequent electrochemical deposition environment preparation uses PH12 as the growth Cu_2O parameter. Figure S3 shows the absorption spectrum of ZnO/Cu_2O prepared by Cu_2O deposition of different thicknesses. If the thickness of Cu_2O grown is too high, it will hinder the transfer of charge carriers and reduce the density of short-circuit current, so it must be transmitted through ultraviolet light.

Therefore, it is necessary to determine the growth time of the electrochemical method through the measurement of ultraviolet light-visible light absorption spectroscopy. When the deposition time was increased to 40 minutes, the light absorption of Cu_2O decreased. This may be because the Cu_2O mentioned above was too thick and the light absorption decreased. Therefore, the subsequent electrochemical method of

Cu_2O uses 30 minutes as the growth time. In order to further identify the quality of Cu_2O crystal prepared by electrochemical method, TEM measurement are performed to determine the integrity of the lattice structure. Before performing TEM measurement, FIB is used as a TEM test piece preparation, as shown in Figure S4(a). Figure S4(b) and Figure S4(c) are the TEM bright-field and dark-field images of Cu_2O, respectively. There is no obvious lattice phase defect. Then the quantitative analysis of the composition is shown in Figure S4(d) -Figure S4(f). The signal in Fig. Figure S4(d) is from Si from the glass substrate; the signal in Figure S4(e) is from Cu from the Cu_2O thin film; the signal in Figure S4(f) is from In of ITO layer. Through the signal analysis of the above three pictures, it can be seen that the Cu_2O crystal is not doped with additional impurities, and Figure S4(g) and Figure S4(h) are the crystal lattice plane of the selected area electron diffraction pattern and the crystal of the high-resolution TEM image. The lattice arrangement shows that the crystal has a high degree of crystallinity, its crystal lattice is complete, and the crystallization characteristics are excellent. Based on the above TEM conclusions, it can be known that the Cu_2O grown by electrochemical method is a single crystal crystal.

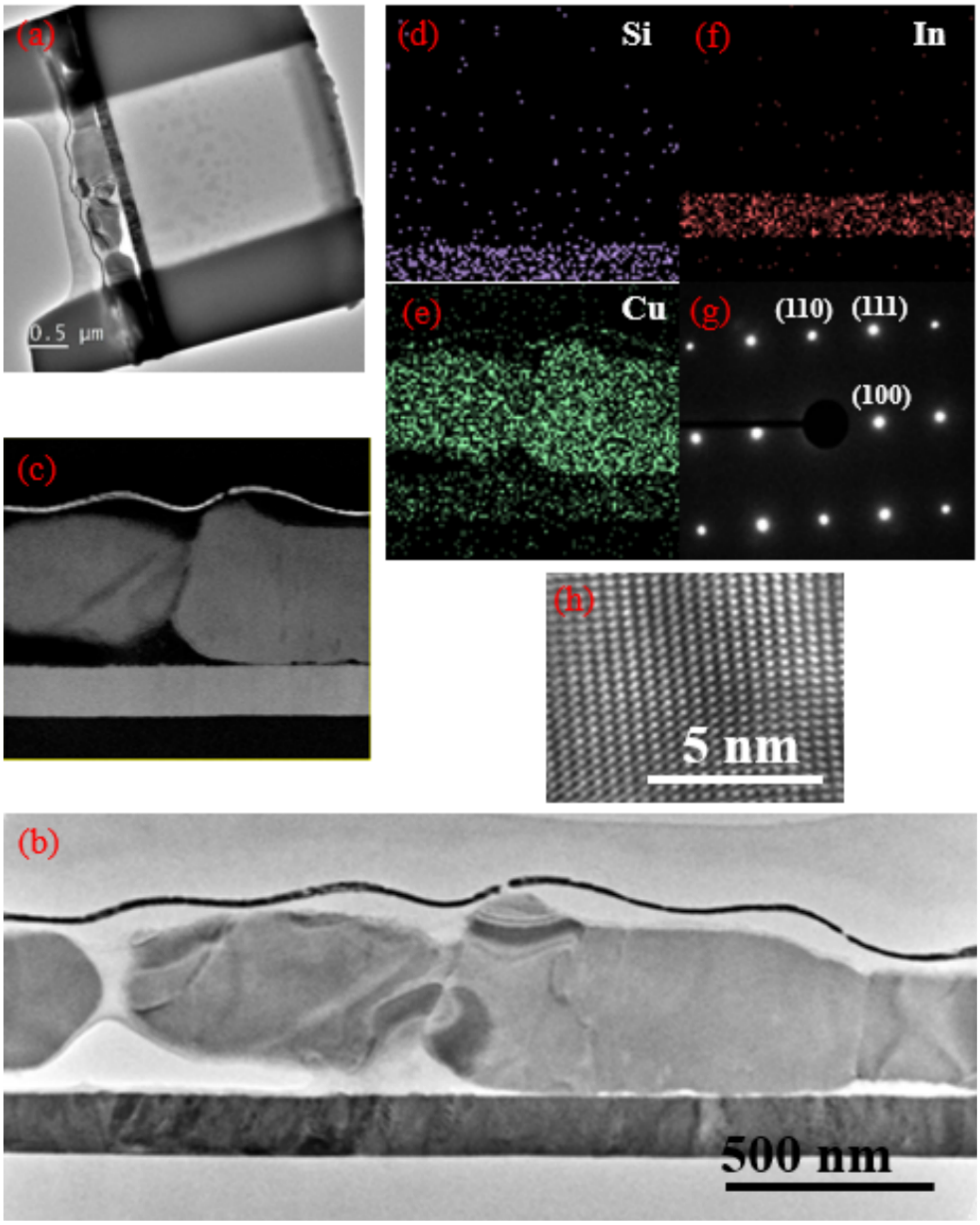

Figure S4. TEM image of Cu_2O single crystal (a) FIB test piece, (b) TEM bright field image, (c) TEM dark field image, (d)-(f) element analysis image, (g) selected area electron winding Schematic, (h) lattice arrangement of high-resolution TEM image

5. CVD growth MoS_2 results

5.1. Multiphoton image analysis results

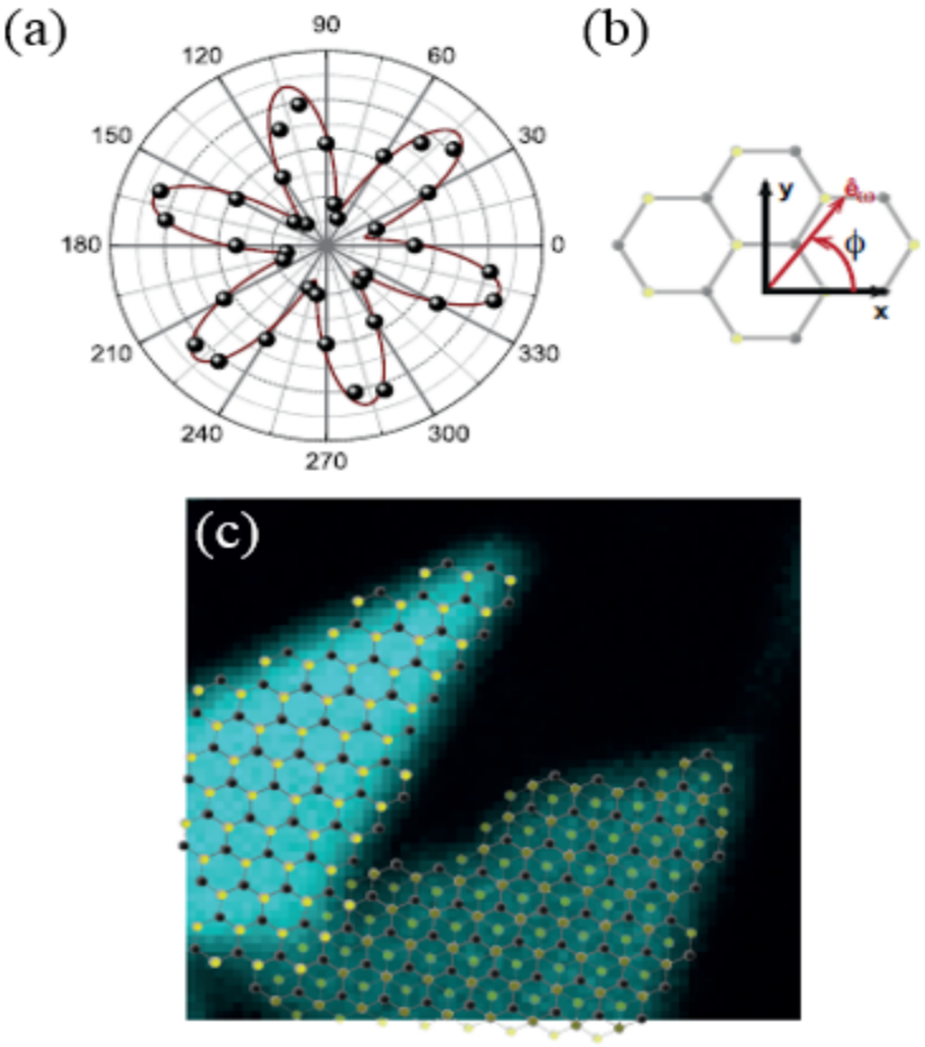

Figure S5.

The sample angle function diagram of the second harmonic intensity of single-layer MoS2, (b) is the top view of the MoS2 crystal orientation relative to the incident light polarization, and (c) is the SH diagram of the sample

Bulk MoS_2 crystal has a triangular prism structure of Bernal stack, which belongs to the $P6_3/mmc$ asymmetric space group, and has an inversion symmetry operation between the two layers of MoS_2. As the number of MoS_2 layers is reduced to a single layer, three atoms can easily form a point group to form a unit cell, so we assign D_3h as a single Layer symmetry (because reverse symmetry is lost in bulk MoS_2); and in even-numbered layer MoS_2, due to the lack of translation symmetry along the Z-axis part, the inversion symmetry operation appears again. The point group of this even-numbered layer is D_3d (even-numbered layers belong to D3d and odd-numbered layers belong to D3h). In this way, the defects caused by the second harmonic in the even-numbered layer MoS_2 can be qualitatively explained. However, due to the inverted symmetry The existence of X^2tensor is equal to zero. Therefore, for even-numbered MoS_2, the second

harmonic will not be generated in the dipole approximation. Due to the symmetry mentioned above, the polarization characteristic provides important crystal information of the MoS_2 atomic layer for double frequency (SHG) analytical measurement. We put the sample on the top of the precision rotating table, the laser wavelength is set at 840nm and focused on the sample, and the sample platform has a fixed linear polarization, so that the sample has a crystalline axis at any angle to the incident polarization. For the SH polarization parallel to the incident light, we set the angle at 5° to measure an SH image of each angle, as shown in Figure S5 [1]. Figure S5(a) shows the measurement results of the polarization dependence of single-layer MoS_2 on SHG. A 6-fold pattern can be clearly observed. Figure S5(b) is the top view of MoS_2 crystal orientation and incident light polarization direction. Figure S5(c) shows the SH images of single-layer and three-layer MoS_2 and the crystal orientation of the crystal lattice obtained from the polarization data in Figure S5(a).

Bulk MoS_2 is composed of two hexagonal lattices of S atoms and triangular prisms occupied by Mo atoms between the S layers. As shown in Figure S6, the number of crystal layers shows different symmetry properties. As mentioned above, the defects caused by the second harmonic in the even number of layers MoS_2 cause X^2 tensor to be equal to zero, which makes the even number of layers MoS_2 will not produce the second harmonic in the dipole approximation. The result of measuring the image signal is much weaker than the odd number of layers, so we can simply analyze the odd and even number of layers through the multiphoton image. The multi-photon image is clearly visible because the two-dimensional film form of MoS_2 breaks the inversion symmetry on the lattice structure. This nonlinear optical effect can be applied to MoS_2 atomic-level thin film materials.

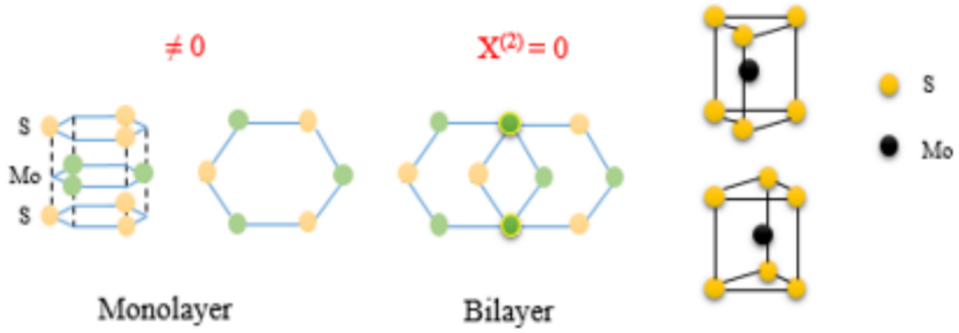

Figure S6.
Schematic diagram of the second harmonic of Inversion asymmetry Inversion symmetry to MoS_2

Although the Raman measurement can also be used to identify the number of MoS_2 layers, the Raman measurement usually depends on the substrate material, which makes the image contrast relatively low. In addition to identifying the advantages of a single-layer film, the second harmonic image can also detect crystals, orientation, single crystal, size and stacking, etc., this method can quickly obtain a complete image of the crystal structure. Figure S7(a) shows the OM image of $MoS_2/SiO_2/Si$, Figure S7(b) and Figure S7(c) shows the second harmonic image, and Figure S7(d) shows the intensity reading of the SHG image data between the two points in Figure S7(b), Figure S7(e) and Figure S7(f) is the OM and SHG images at different positions, Figure S7(g) is the intensity reading of the SHG image, Figure S7(f) shows the data between two points. The signal of the x-axis position in the 150 area is at least 60% lower than that in the 100 and 200 areas. This means that the double-numbered layer MoS_2 mentioned above does not produce second harmonics in the dipole approximation. So the analysis results shows Very low second harmonic signal, and the small signal observed around the strong signal comes from the boundary of the MoS_2 layer. The multiphoton image also has a special function.

It can also be color-coded according to the crystal orientation of the material itself, such as as shown in Figure S7(h).

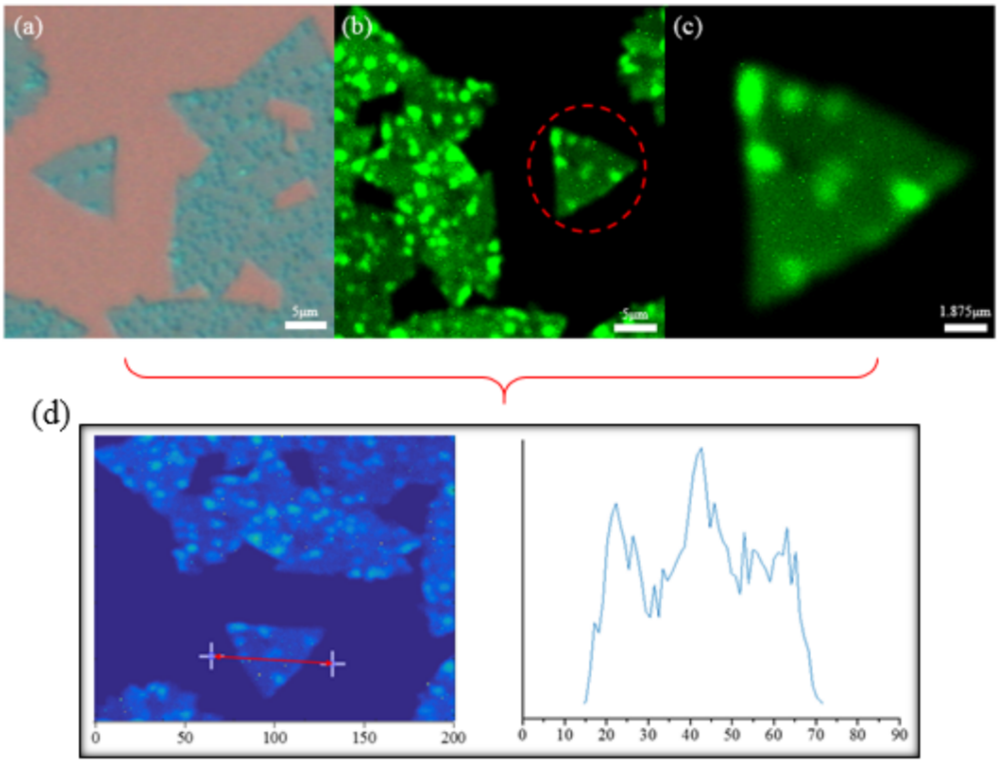

Figure S7. The OM image under $MoS_2/SiO_2/Si$, (b) and (c) are the comparison of the multiphoton image of (a) (the multiphoton image is opposite to the left and right sides of the OM image), and (d) is the reading taken (b) the SHG image of the intensity data between the two points, (e) the OM image at different locations, (f) the multiphoton image comparison of (e), and (g) the reading of the two points (f) SHG image of the intensity data between the two, (h) is the multi-photon image of different color coding

5.2. SEM analysis

A single-layer MoS_2 film is grown using CVD and the size of which is about 20-30μm. When argon gas is injected at 100sccm, a uniformly contrasted triangular or star-shaped two-dimensional MoS_2 structure can be seen through the OM image. The deposit is shown in Figure S8. The upper right small image is a SEM image. The MoS_2 structure grown by the CVD method covers a large area, indicating that the amount of argon gas of 100sccm is sufficient for the transmission of S and MoO_3 vapors. The MoS_2 film structure is formed on the SiO_2/Si substrate by adjusting the heating and holding time.

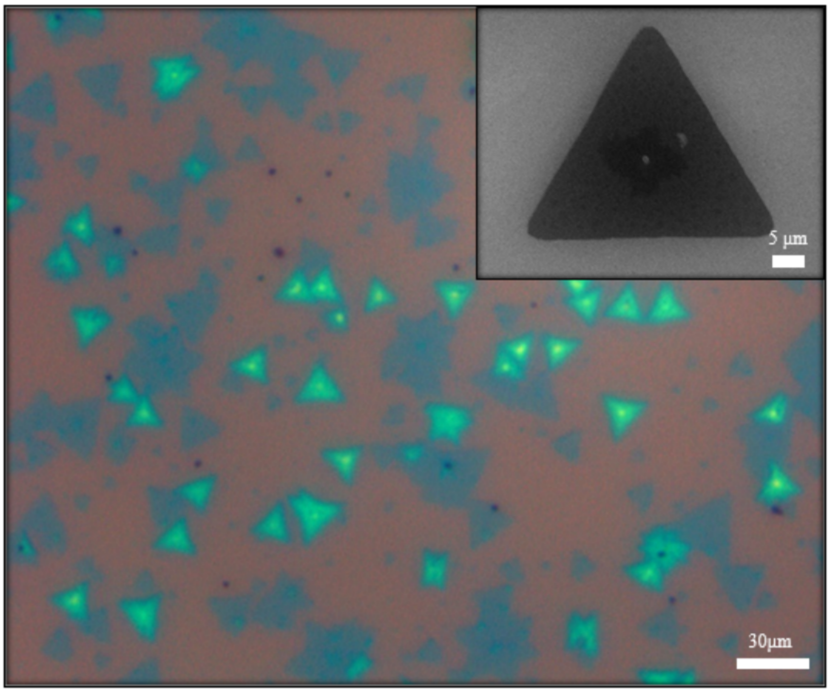

Figure S8. The OM and SEM images of MoS_2 grown by CVD method

5.3. Raman and PL analysis

In order to understand the optical phenomena between different layers of molybdenum disulfide Raman spectrum is employed as shown in Figure S9, where the X axis represents the Raman Shift and the Y axis represents the Raman signal It can be found that there are peaks near the Raman shifts of 383cm^{-1} and 403^{-1}cm. These two peaks represent E_{12g} mode and A_{1g} mode, respectively. Among them, in-of-plane (E_{12g}) is due to the two S atoms and Mo atoms of MoS_2 oscillating in opposite directions, while out-of-plane (A_{1g}) only oscillates in opposite directions by sulfur atoms.As shown in Figure S9(c), when the peak difference (k) is less than 20, it can be regarded as a single layer. Figure S9(a) shows the peak sizes of E_{12g} and A_{1g} corresponding to layers 1-4, and the magnitude of the difference between layers 1-4. As described in the previous chapter, the number of layers can be determined by the difference between E_{12g} mode and A_{1g} mode, and then the distribution position of each layer in the image can be known. When MoS_2 increases from a single layer to multiple layers, the Van der Waals force between layers increases, and the restore force of atoms will also be affected. It can be expected that E_{12g} mode and A_{1g} mode will have blue shift with the increase of the number of layers. A_{1g} mode has such a trend, but E_{12g} mode moves in the opposite direction (red shift), which reflects that it is affected by additional interlayer interaction. It is because MoS_2 changes the internal structure with the increase of the number of layers, which affects the stacking of MoS_2, so that the Coulomb force of the layers in a large range causes the E_{12g} mode to move in the opposite direction. Therefore, we can judge the number of molybdenum disulfide layers in this area by the difference between A_{1g}-E_{12g} as shown in Figure S9(a). Figure S9(b) is the PL luminescence spectrum of MoS_2. The 532nm wavelength band is used as the laser excitation light source in the measurement. The spectrum consists of two peaks, corresponding to A1 exciton and B1 exciton respectively. At 667nm (1.86eV) and 627nm (1.97eV), the A1 exciton and B1 exciton have direct exciton transitions that are separated from the valence band spin-orbital coupling. The K point of the direct exciton transition of these two resonances in the Brillouin zone has been confirmed. Generally speaking, the peak of MoS_2 luminescence is about 667nm, which

is converted to an energy of 1.86eV, which is the direct energy gap of the MoS_2 film. The above data results show that the MoS_2 we have grown is a single-layer structure.

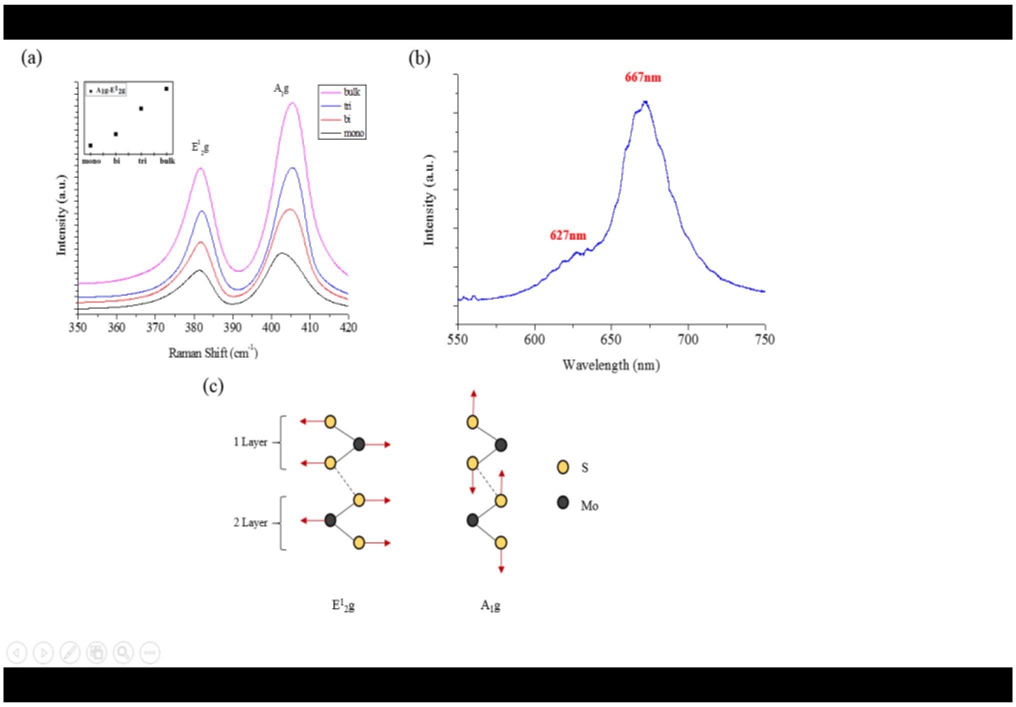

Figure S9. The analysis results of MoS_2 grown by CVD, (a) Raman spectrum of single-layer, double-layer, triple-layer and Bulk shape,(b) PL measurement result of single-layer MoS2, the emission band is at 667nm, converted energy 1.86eV, (c) Schematic diagram of E12$_g$ and A_{1g} atomic modes of single layer and double layer

5.4. AFM analysis

The thickness of the MoS_2 film can be determined through AFM measurement. The surface morphology and height profile results are shown in Figure S10(a) and Figure S10(b), respectively. The surface morphology of SiO_2 and MoS_2 can be clearly seen in Figure S10(a), and Figure S10(b) shows that the height profile of the cross-section line in Figure S10(a) shows that the grown MoS_2 is in SiO_2. The thickness on the /Si substrate is 0.72nm, and the measurement result can be regarded as a single-layer MoS_2 structure.

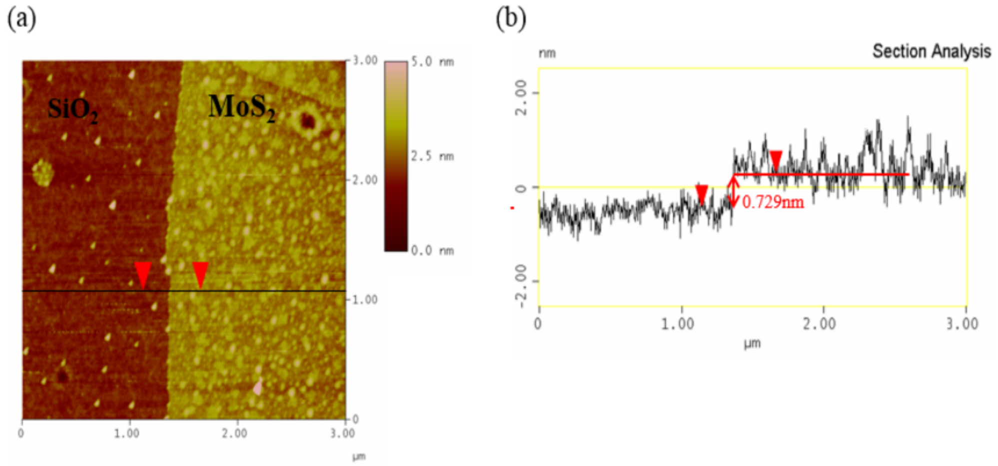

Figure S10. AFM image of CVD grown MoS_2, (a) is the surface morphology of SiO_2 and MoS_2, (b) is the result of (a) the height profile of the cross section

As shown in Figure S11, the blue line, the black line and the red line are the PL emission spectra of MoS_2, Cu2O and MoS_2/Cu_2O, respectively. In the MoS_2 spectrum by two peaks, corresponding to the A1 exciton and B1 exciton are 667nm (1.86eV) and 627nm (1.97eV), respectively. When we measured the PL of Cu_2O, we found that the luminescence band of Cu_2O grown by electrochemical method was about 720 to 900nm.

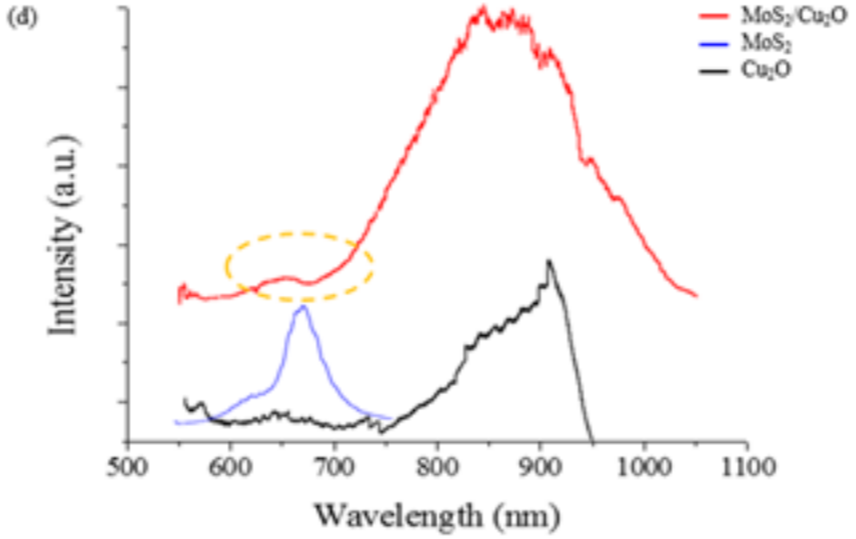

Figure S11. The blue, black, and red lines are the PL emission spectra of MoS_2, Cu_2O, and MoS2/Cu2O, respectively.

6. Analysis of Structural Characteristics of Photoelectrochemical Biosensors

6.1. X-ray diffractometer analysis

Cu_2O is sensitive to the pH value of the deposition environment, and this characteristic can be used to indirectly control its preferred growth lattice plane. Usually, the (111) or (200) lattice plane can be optimized by controlling the deposition environment by pH value. The cuprous oxide sample grown by electrochemical method was detected by X-ray diffraction analysis to verify whether it grew into a biological wafer meeting the (111) lattice plane required for this study. The experimental results are shown in Figure S1. In the figure, it can be observed that the lattice diffraction peaks of cuprous oxide appear at 2=29.4°, 36.3°, 42.2°, 61.2°, and 73.4°, which are compared from the Joint Committee on Powder Diffraction Standards (JCPDS) card (110), (111), (200), (220), (311) lattice directions. It can be seen from the results that the grown cuprous oxide film is a biochip film with good light response required for this study.

6.2. Hall effect measurement and analysis

In photoelectrochemical biosensors, if the material has high saturation electron velocity and electron mobility, the effective carrier concentration will be high thereby more photocurrent can be collected. Before measuring, we need to know that the average diameter (D) of the joint. The thickness of the sample (d) must be smaller than the distance (L) between the joints. The film thickness was determined to be approximately 2.1 μm by SEM cross-section in Figure S12. The results show that the carrier type of the material is P-type, the carrier mobility is $9.77*10^3(cm^2/V{\cdot}s)$, the resistivity is $2.89*10^{-3}(\Omega{\cdot}cm)$,carrier concentration 2.2*1017(cm^{-3}).

6.3. Ultraviolet-Visible Absorption Spectroscopy

We can observe from Figure S13 that the main light absorption band of cuprous oxide is largest in the range of 300nm - 580nm, and its corresponding wavelength light source is from near violet light to green light, accounting for about half of the wavelength range of visible light. The ultimate purpose of this study is to apply it

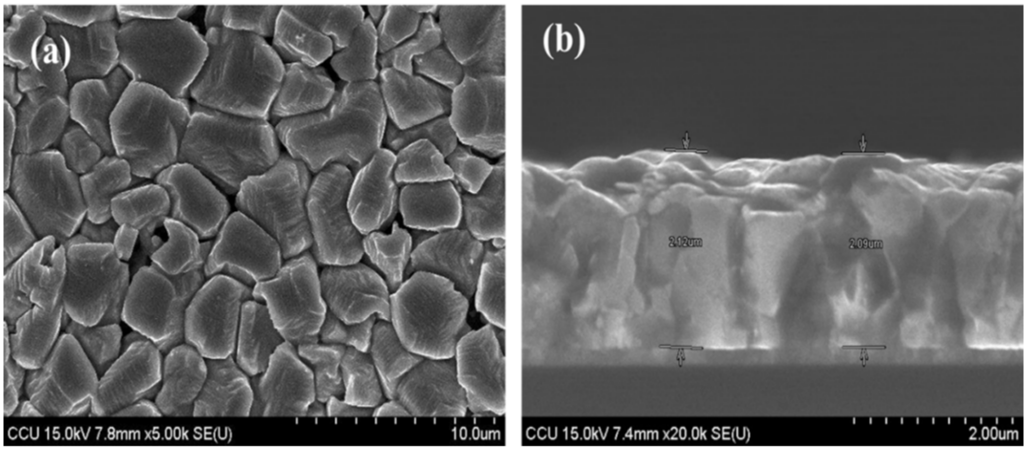

Figure S12. Caption:

in actual detection, so this experiment uses the most common white light source to complete the experimental light source. Although half of the wavelength cannot be absorbed, we use the above-mentioned material structural characteristics to improve the light response to improve the energy lost to the half of the spectrum that fails to absorb.

This study used an interdigitated microelectrode, a design capable of generating p-DEP force on each cell of the electrode when a high-frequency sine wave signal was applied to the electrode. Under the influence of the p-DEP force, these cells will be attracted to the higher potential region of the electric field. The applied cells can be captured on the tips of the electrodes, and after the cells are concentrated, the cell impedance on the biosensing wafer can be measured. In the study, because the dropped cells will float in the solution, it is necessary to wait five minutes for the cells to settle to the bottom of the wafer. In order to make the cells produce p-DEP effect, before measuring A549, H460, H520, a 1 MHz, 10Vp-p sine wave was applied to the electrode for 10 minutes, so that there was enough time to concentrate the cells. Cell measurements were then performed at 4 kHz, the frequency at which the observed signal had the largest and stable response in the bioimpedance sensor. Cancer cells suspended on the biochip were driven by p-DEP and attracted to the tips of the microelectrodes. DEP manipulation leads to an increase in the sensing sensitivity of the biochip due to the significant increase in the electrode-solution interface impedance. Next, the conducting and capacitive components of the chip are calculated by measuring the real and imaginary parts of the admittance

Author Contributions: Conceptualization, W.S.F. and S.Y.H.; methodology, H.-T.N.; software, H.-T.N.; validation, W.T. H. and H.-C.W.; formal analysis, W.S.F.; investigation, W.T. H.; resources, W.H.C.; data cura-tion, W.S.F. and S.Y.H.; writing—Original draft preparation, A.M, H.-T.N.; writing—Review and editing, A.M, F.C. L. and H.-C.W.; supervision, H.-C.W.; project administration, H.-C.W. All authors have read and agreed to the published version of the manuscript.

Conflicts of Interest: The author declare no conflict of interest.

Funding: This research was supported by the Ministry of Science and Technology, The Republic of China under the grants MOST 105-2923-E-194-003 MY3, 108-2823-8-194-002, 109-2622-8-194-001-TE1, and 109-2622-8-194-007. This work was financially/partially supported by the Advanced Institute of Manufacturing with High-tech Innovations (AIM-HI) and the Center for Innovative Research on Aging Society (CIRAS) from The Featured Areas Research Center Program within the frame-work of the Higher Education Sprout Project by the Ministry of Education (MOE) in Taiwan.

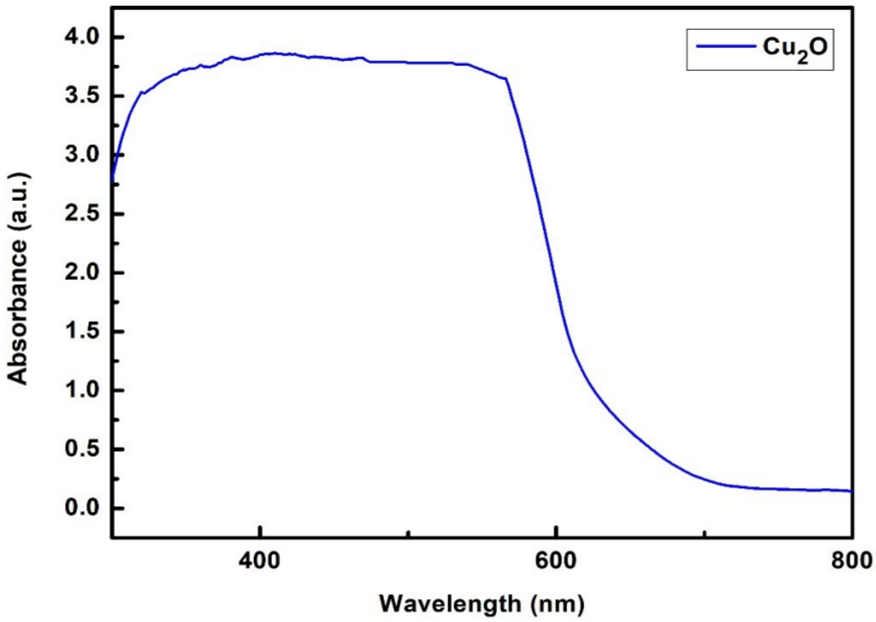

Figure S13. UV-Vis spectrum analysis of Cu_2O

References

1. Malard, L.M.; Alencar, T.V.; Barboza, A.P.M.; Mak, K.F.; De Paula, A.M. Observation of intense second harmonic generation from MoS 2 atomic crystals. *Physical Review B* **2013**, *87*, 201401.
